# Evaluation of the PE*Δ*III-LC3-KDEL3 Chimeric Protein of *Entamoeba histolytica-*Lectin as a Vaccine Candidate against Amebic Liver Abscess

**DOI:** 10.1155/2021/6697900

**Published:** 2021-03-20

**Authors:** Sandra L. Martínez-Hernández, Viridiana M. Becerra-González, Martín H. Muñoz-Ortega, Víctor M. Loera-Muro, Manuel E. Ávila-Blanco, Marina N. Medina-Rosales, Javier Ventura-Juárez

**Affiliations:** ^1^Departamento de Morfología, Centro de Ciencias Básicas, Universidad Autónoma de Aguascalientes, C. P. 20131., Aguascalientes, Ags., Mexico; ^2^Departamento de Química, Centro de Ciencias Básicas, Universidad Autónoma de Aguascalientes, C. P. 20131., Aguascalientes, Ags., Mexico; ^3^Departamento de Fisiología y Farmacología, Centro de Ciencias Básicas, Universidad Autónoma de Aguascalientes, C. P. 20131., Aguascalientes, Ags., Mexico

## Abstract

*Entamoeba histolytica* is an intestinal parasite that causes dysentery and amebic liver abscess. *E. histolytica* has the capability to invade host tissue by union of virulence factor Gal/GalNAc lectin; this molecule induces an adherence-inhibitory antibody response as well as to protect against amebic liver abscess (ALA). The present work showed the effect of the immunization with PE*Δ*III-LC3-KDEL3 recombinant protein. *In vitro*, this candidate vaccine inhibited adherence of *E. histolytica* trophozoites to HepG2 cell monolayer, avoiding the cytolysis, and in a hamster model, we observed a vaccine-induced protection against the damage to tissue liver and the inhibition of uncontrolled inflammation. PE*Δ*III-LC3-KDEL3 reduced the expression of TNF-*α*, IL-1*β*, and NF-*κ*B in all immunized groups at 4- and 7-day postinfection. The levels of IL-10, FOXP3, and IFN-*γ* were elevated at 7 days. The immunohistochemistry assay confirmed this result, revealing an elevated quantity of +IFN-*γ* cells in the liver tissue. ALA formation in hamsters immunized was minimal, and few trophozoites were identified. Hence, immunization with PE*Δ*III-LC3-KDEL3 herein prevented invasive amebiasis, avoided an acute proinflammatory response, and activated a protective response within a short time. Finally, this recombinant protein induced an increase of serum IgG.

## 1. Introduction

*Entamoeba histolytica* is an enteric protozoan parasite and the etiologic agent of amebiasis [1]. This disease is a worldwide health problem that affects an estimated 50 million people and causes over 100,000 deaths annually (primarily in developing countries) [2]. Children exposed to repeated infections can suffer malnourishment and the stunting of growth [3]. Whereas about 90% of amebic infections are asymptomatic, the other 10% display a spectrum of diseases: acute diarrhea, dysentery, amebic colitis, and amebic liver abscess (ALA); the latter is the most common extraintestinal manifestation of amebiasis [4], triggered by the capacity of *E. histolytica* to produce host cell death and a destructive inflammatory response [5]. Invasive amebiasis is treated with nitroimidazoles, which have toxic side effects and require complementary drugs to cure the infection in 40–60% of patients [6]. Despite the medical importance of this parasite, an effective vaccine to prevent amebiasis has not yet to become available. In the search for alternative treatments, the amebic galactose-binding lectin is today among the most commonly used antigens for carrying out exploratory assays. This complex protein is located on the surface of the parasite and consists of three subunits. The main component, the heavy subunit of 170 kDa, is one of the most immunogenic *E. histolytica* molecules [7], bearing a carbohydrate recognition domain rich in cysteines (LC3) [8].

In one of our previous studies, a chimeric vaccine (PE*Δ*III-LC3-KDEL3) was elaborated that contained the *Entamoeba histolytica* LC3 fragment fused to domains I and II of exotoxin A of *Pseudomonas aeruginosa* (*P. aeruginosa*) and the carboxy-terminal signal KDEL3 [9]; this chimeric molecule was evaluated in capability to raise antibodies in sera from animals immunized; it was detected a raised serum IgG at 2.03- to 2.1-fold greater concentration in immunized versus nonimmunized animals [9]. The aim of the present study was to evaluate the effect of PE*Δ*III-LC3-KDEL3 as a recombinant vaccine through analysis of its immunogenic activity (antibody production and modulation of inflammation), inhibition of cytotoxicity, and protection against the development of ALA.

## 2. Materials and Methods

### 2.1. Animals

Male golden hamsters (*Mesocricetus auratus*) weighing 80-100 g were used in this study. The animals were dewormed by ivermectin 5 mg in 500 ml of distilled water during the first week, after that, were maintained on standard diet with drinking water *ad libitum*. All animals received human care according to the guidelines of the Committee on Bioethics in the animal facilities of the Autonomous University of Aguascalientes, Aguascalientes, Mexico, which is based on the guidelines for animal research published by the National Institute of Health (National Research Council (US) Committee for the Update of the Guide for the Care and Use of Laboratory Animals, 2011) and the Mexican Official Norm: NOM-062-ZOO-1999 [10].

### 2.2. *E. histolytica* Culture

*E. histolytica* HM-1:IMSS trophozoites were grown axenically in TYI-S-33 [11]. Trophozoites were harvested at 72 h for use in all experiments.

### 2.3. Vaccine Antigen PE*Δ*III-LC3-KDEL3

Recombinant vaccine was designed, purified, and analyzed by Martínez-Hernández et al. [9] and detected with a rabbit polyclonal anti-6X His tag antibody (Ab1187, Abcam, Cambridge, UK) and a rabbit polyclonal antibody monospecific to *E. histolytica* [12].

### 2.4. Immunization

Hamsters were divided into five groups (*n* = 5 each), intact and sham as a control and three experimental. The former was healthy intact (no treatment nor infection) and sham, administered the vehicle (sterile PBS 1x, 100 *μ*l) intramuscularly with a 0.40 mm × 1/2 inch needle. The three experimental groups were immunized intramuscularly with PE*Δ*III-LC3-KDEL3 at doses of 50, 75, and 100 *μ*g/animal. The PE*Δ*III-LC3-KDEL3 and the vehicle were applied on days 1, 7, and 14.

### 2.5. Experimental Hepatic Amebiasis

One week after the last immunization, amoebic liver abscesses were induced by direct hepatic inoculation as previously described [13]. On day 4 or 7 postinfection, the animals were anaesthetized with sodium pentobarbital and sacrificed. To evaluate the development of ALAs, liver samples were taken from all the animals and fixed with 4% paraformaldehyde processed in paraffin and submitted to hematoxylin and eosin (H&E) staining. The tissue sections were stored in RNA later at -81°C to await analysis.

### 2.6. Antibody Detection by ELISA

Serum was examined *in vitro* by ELISA for the identification of antibodies against *E*. *histolytica* elicited by treatment with PE*Δ*III-LC3-KDEL3, by a slightly modified method [14], using *E. histolytica* membrane protein antigen [12], then, HRP-conjugated anti-hamster IgG antibody (H1643, SIGMA, San Luis Missouri, USA) (1 : 1000). After that, it was developed with orthophenylendiamine (Thermo Scientific 34005, MA USA) and read at 490 nm on an iMark-microplate-reader (Bio-Rad, Hercules, California, USA).

### 2.7. Cytotoxicity Assay

2 × 10^4^ trophozoites of *E. histolytica* were resuspended in TYI-S-33 medium; they were treated with dilutions (1 : 100) of immune serum (from the 50, 75, and 100 *μ*g groups) and sham serum at 37°C for 1 h. For a positive control, trophozoites were left untreated. Simultaneous with the preincubation, 2 × 10^5^ HepG2 cells were seeded in 24-well plates. Once they get confluence, trophozoites were added, and interaction between HepG2 cells and *E. histolytica* took place at 37°C for 2 h. After that, wells were washed, fixed with 2% PFA, stained with 0.1% methylene blue in 10 mM borate buffer for 15 min, and finally washed 3 times. Subsequently, 0.1 M HCl was added, and each well was left at 37°C for 30 min to extract the stain. Absorbance was then read on a spectrometer at 655 nm (OD_655_). The percentage of monolayer destruction was calculated as follows: [(OD_655_ of control wells)–(OD_655_ of experimental wells)/(OD_655_ of control wells)] × 100.

### 2.8. Analysis of Cytokine Expression by RT-qPCR

The RNA extracted from the liver tissue from the immunized and nonimmunized animals was analyzed by RT-qPCR using specific primers for cytokine genes (Table 1). Total RNA was isolated from 100 mg of liver tissue of control and experimental animals using the SV Total RNA Isolation System (Z3100, Promega, Madison, Wisconsin, USA), according to the manufacturer's protocol, then quantified with a Biodrop (Biochrom, Waterbeach Cambridge, United Kingdom) and stored at -80°C until needed. Reverse transcription was performed with 1 *μ*g of total RNA and the GoScript Reverse Transcription System (A5001, Promega, Madison, Wisconsin, USA). Quantitative PCR was carried out with the Maxima SYBR Green/ROX qPCR Master Mix (2x) (K0221, Thermo Fisher Scientific, Waltham, Massachusetts, USA) in a StepOne System (Applied Biosystems, Foster City, California, USA), utilizing the following programming: 50°C for 2 min, 95°C for 3 min, 40 cycles of 95°C for 45 sec, and 60°C for 35 sec. Oligonucleotides were designed to target the cytokine genes. Relative expression was normalized to that of *β*-actin, and the differences were determined using the 2^-*ΔΔ*Cq^ relative method [15].

### 2.9. Immunohistochemistry

IFN-*γ*-positive cells and *E. histolytica* trophozoites were identified in liver tissue by immunohistochemistry as done by Ventura-Juárez et al. [16], Briefly, we used primary rabbit polyclonal anti-IFN-*γ* antibody (500-P32, Pepro-Tech, Cranbury, NJ, USA) diluted 1 : 200, 1 h at 37°C. As a secondary antibody, Dako Envision system AP (IgG rabbit-mouse, K4065, DAKO, DNK) for 2 h and peroxidase activity were developed with diaminobenzidine for 5 min. Images were captured and analyzed with the Image Pro Plus Software 4.5.1 (Media Cybernetics, Bethesda, Maryland, USA) in a Zeiss Axioscop 40/40L microscope (Zeiss, Oberkochen, DEU).

### 2.10. Statistical Analysis

Differences between groups were assessed with one-way analysis of variance (ANOVA), followed by Tukey's and Dunn's *post hoc* test, using GraphPad Prism version 8.0 for Windows (GraphPad Software, San Diego, California, USA). Data are expressed as the mean ± SEM of the values from three independent experiments. Significance was considered at *p* < 0.05.

## 3. Results

### 3.1. PE*Δ*III-LC3-KDEL3 Increased Serum Antibody Levels

**Figure 1:**
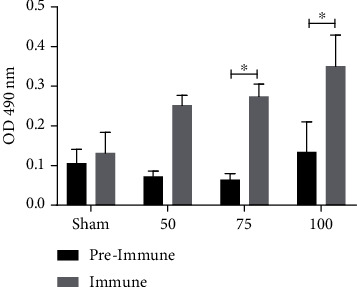
The PE*Δ*III-LC3-KDEL3 vaccine induced IgG antibody production. Serum samples were evaluated by ELISA. Bars represent the mean ± SEM of three independent assays. Statistical analysis was performed with one-way ANOVA ^∗^(*p* < 0.05).

**Figure 2:**
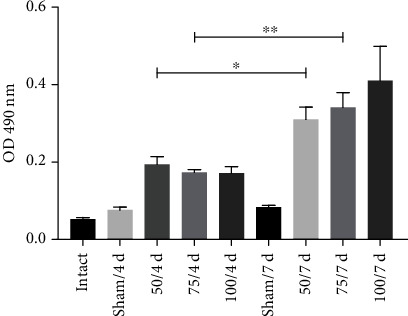
The antibody levels were elevated in the animals receiving the PE*Δ*III-LC3-KDEL3 vaccine even in the postinfection period.

The levels of the specific antibodies elicited by the recombinant vaccine were determined in the serum from animals before and after immunization. All immunized hamsters developed a greater quantity of IgG antibodies than nonimmunized animals (Figure 1), although the difference was significant only for the 75 and 100 *μ*g groups. Immunized hamsters were infected 7-day postimmunization, then sacrificed at 4- and 7-day postinfection. Compared to the sham animals at day 7 postinfection, the antibody level in the 50 and 75 *μ*g groups showed a significant increase, while the level in the 100 *μ*g group was not significantly higher (Figure 2).

### 3.2. Immune Sera Inhibit *E. histolytica* Cytopathic Activity on HepG2 Cells

**Figure 3:**
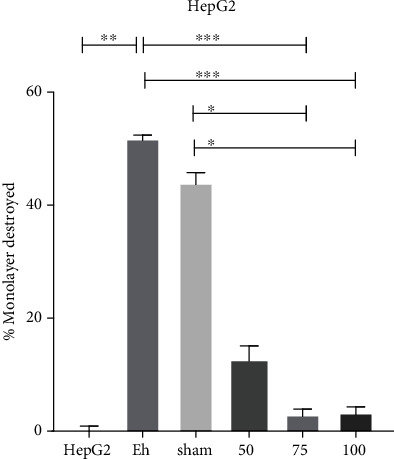
Inhibition of cytopathic effect of *E. histolytica* on HepG2 cells. Serum from the immunized groups inhibits destruction. Serum from sham animals generated a high percentage of destruction, similar to the positive control. HepG2 cells not exposed to *E. histolytica* represented the negative control. Data are expressed as the mean ± SEM of three independent assays, no parametric test Dunn's post hoc. ^∗^*p* < 0.05; ^∗∗^*p* < 0.01; ^∗∗∗^*p* < 0.001.

The ability of *E. histolytica* trophozoites to recognize and adhere to cells leads to cell death followed by phagocytosis [17]. Thus, HepG2 liver cells were herein exposed to trophozoites previously incubated for 1 h with the serum from immunized or nonimmunized animals. The virulent trophozoites without pretreatment and pretreated with serum from nonimmunized hamsters generated a high percentage of destruction of HepG2 cells (51% and 44%, respectively). The trophozoites pretreated with the serum from immunized animals inhibit destruction of the HepG2 cell monolayer, being 12.3% with 50 *μ*g and only 3.0% with both 75 and 100 *μ*g (Figure 3). This indicate that antibodies from vaccine-immunized hamsters recognized the LC3 fragment of trophozoites and interfered with the ability of the parasite to bind to and destroy the HepG2 cells.

### 3.3. Gene Expression of TNF-*α*, IL-1*β*, IFN-*γ* NF-*κ*B, FOXP3, and IL-10 in Vaccinated Hamsters

**Figure 4:**
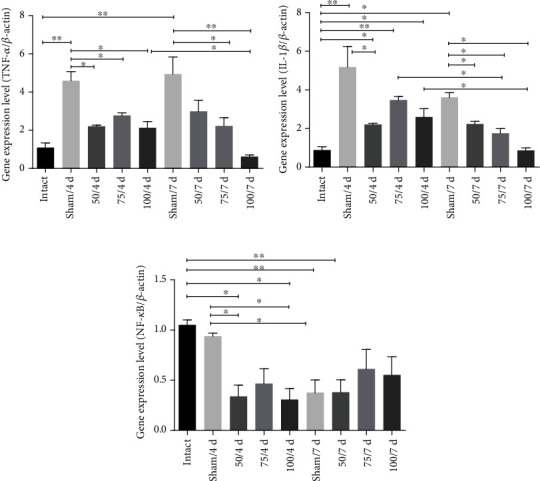
The vaccine reduced the expression of the proinflammatory genes. (a, b) TNF-*α* and IL-1*β* in all immunized hamsters at 4- and 7-day postinfection were downregulated. (c) The NF-*κ*B gene expression was also diminished in all immunized animals. Comparisons among groups: ^∗^*p* < 0.05 and ^∗∗^*p* < 0.01.

Compared to the sham group, the gene expression for the proinflammatory cytokine TNF-*α* was significantly lower at 4- and 7-day postinfection for the hamsters receiving 50, 75, and 100 *μ*g (*p* < 0.05, *p* < 0.01) is important to realize that 100 *μ*g at 7 days was even more low than at 4 days (Figure 4(a)). The IL-1*β* gene expression was diminished at 4 and 7 days in the 50 *μ*g group (*p* < 0.05); likewise, gene expression of this cytokine was low at 75 and 100 *μ*g at 7 days compared those to 4 days. Anterior results show a diminish timeline of the IL-1*β* gene expression (Figure 4(b)). To corroborate these results, the gene expression for nuclear factor kappa B (NF-*κ*B) was also examined, finding it to be downregulated in all immunized hamsters (*p* < 0.05, *p* < 0.01), except 75 and 100 *μ*g at 7 days (Figure 4(c)).

**Figure 5:**
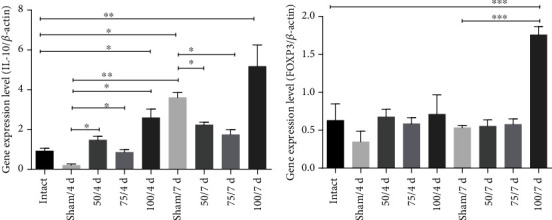
The PE*Δ*III-LC3-KDEL3 vaccine upregulated IL-10 and FOXP3. (a, b) The 100 *μ*g concentration of the vaccine induced a greater IL-10 and FOXP3 gene expression at 7-day postinfection. (b) Comparisons between groups: ^∗^*p* < 0.05, ^∗∗^*p* < 0.01, and ^∗∗∗^*p* < 0.001.

**Figure 6:**
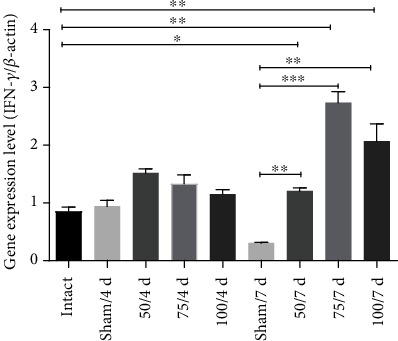
The PE*Δ*III-LC3-KDEL3 vaccine promoted the expression of IFN-*γ*. The IFN-*γ* gene expression in the 50, 75, and 100 *μ*g groups was only elevated at 7-day postinfection. Comparisons between groups: ^∗^*p* < 0.05, ^∗∗^*p* < 0.01, and ^∗∗∗^*p* < 0.001.

IL-10 participates in downregulating the inflammatory process. The gene expression of IL-10 increases at all doses at 4 days (*p* < 0.05 and *p* < 0.01, respectively) compared to sham (Figure 5(a)). No significant difference existed among immunized groups (50, 75, and 100 *μ*g) and intact hamsters, at 7 days only increase IL-10 gen in sham and 100 *μ*g (Figure 5(a)). Regarding FOXP3, the gene expression increased only in 100 *μ*g at 7-day postinfection (*p* <0.001) (Figure 5(b)). Additionally, the expression of INF-*γ* gene in all immunized animals was significantly increased until 7-day postinfection (*p* < 0.05, *p* < 0.01, *p* < 0.001) (Figure 6).

**Figure 7:**
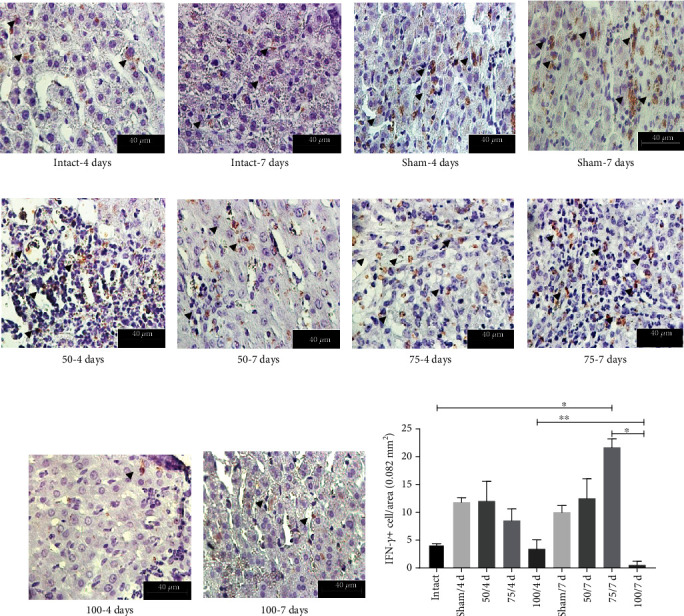
Immunohistochemical analysis of +IFN-*γ* hepatic cells. Original magnification 400x. (a–j) Based on the immunohistochemistry assay, the quantity of +IFN-*γ* cells was determined in the liver tissue of hamsters (arrowhead). (k) The cells were abundant in 75 *μ*g group at 7 days.

The immunohistochemistry assay confirmed this result, revealing an elevated quantity of +IFN-*γ* cells in liver tissue from animals immunized with 75 *μ*g (Figures 7(g), 7(h), and 7(k)). As can be appreciated, the PE*Δ*III-LC3-KDEL3 vaccine had a downregulatory influence on the hamster immune response, which included anti-inflammatory effects.

### 3.4. Effects of the PE*Δ*III-LC3-KDEL3 Vaccine on ALA Formation

**Figure 8:**
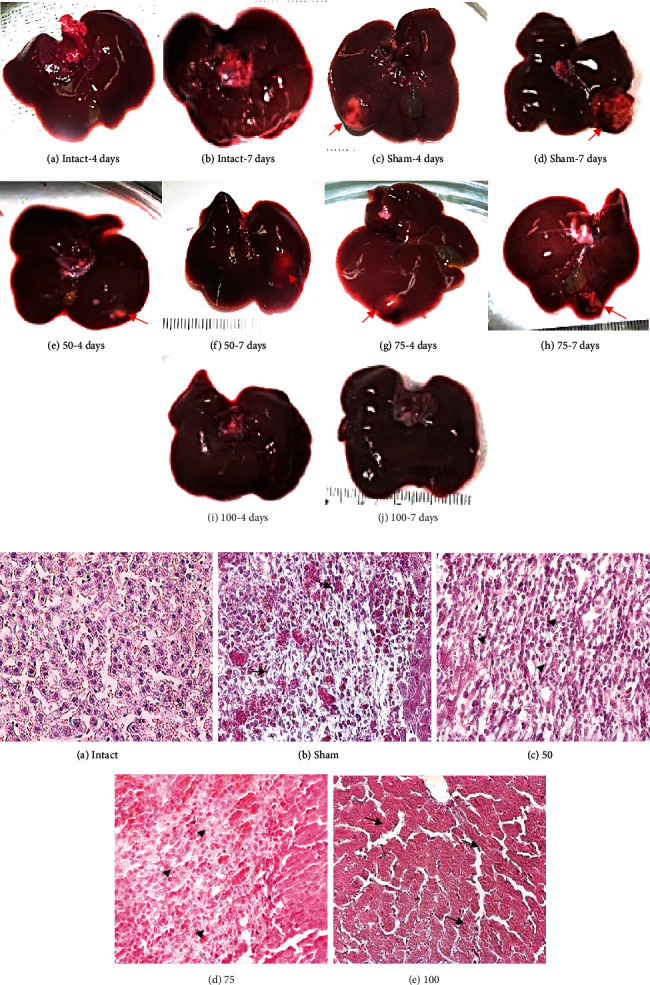
(a) Macroscopic analysis of amebic liver abscess formation. (A, B) No abscesses were detected in the liver of uninfected hamsters. (C, D) The liver of the sham group showed the characteristic ALA lesions. (E, F) The 50 *μ*g group exhibited smaller lesions on day 7 than day 4 than sham. (G, H) A single small lesion is seen in the 75 *μ*g group (arrows). (I, J) No lesion was detected in the liver of the 100 *μ*g group. (b) Light microscopy of paraffin histological technique of amoebic liver abscess. Original magnification 400x. (A) In the intact hamsters, normal architecture was found in the liver tissue. (B, C) In two of the infected groups, sham and vaccinated with 50 *μ*g, a necrotic area was observed in the liver parenchyma, accompanied by inflammatory infiltrate (arrows, asterisk). (D) In the 75 *μ*g group, the parenchyma displayed no tissue necrosis (arrowhead). (E) In the 100 *μ*g group, the parenchyma was similar to the intact animals, and there was no tissue necrosis or inflammation (arrows, cross).

Male hamsters were inoculated with 5 × 10^5^ virulent *E. histolytica* trophozoites 7 days after vaccination. The liver tissue of the sham group showed characteristic ALA lesions: large, pale hemorrhagic zones located at the site of inoculation and were larger on 7 days than 4 days (Figures 8(a), (D) and 8(a), (C)). Compared to the sham hamsters, a considerable decrease in liver damage was found in all immunized groups; the liver tissue of the 50 *μ*g group displayed a small white lesion without hemorrhagic borders at 4 and 7 days (Figures 8(a), (E) and 8(a), (F)). The liver from inoculated with 75 *μ*g group exhibited a small lesion (at 4 days Figure 8(a), (G)) with a hemorrhagic area (at 7 days) (Figure 8(a), (H)). Surprisingly, evaluation of the liver tissue of the 100 *μ*g group revealed the absence of any lesion or hemorrhagic areas (at days 4 and 7; Figures 8(a), (I) and 8(a), (J)). Histological analysis evidenced necrosis of the liver parenchyma (asterisks) and inflammatory infiltrate in the sham (arrows in Figure 8(b), (B)), and in 50 and 75 *μ*g groups, contrarily, there was a better architecture of the parenchyma without any tissue necrosis, although inflammatory infiltrate was detected (arrowheads, Figures 8(b), (C) and 8(b), (D)). Surprisingly, the architecture of the liver parenchyma was similar for the 100 *μ*g (Figure 8(b), (E)) and intact group (Figure 8(b), (A)), with no tissue disruption or inflammatory infiltrate.

### 3.5. Detection and Quantification of *E. histolytica* Trophozoites by Immunohistochemistry

**Figure 9:**
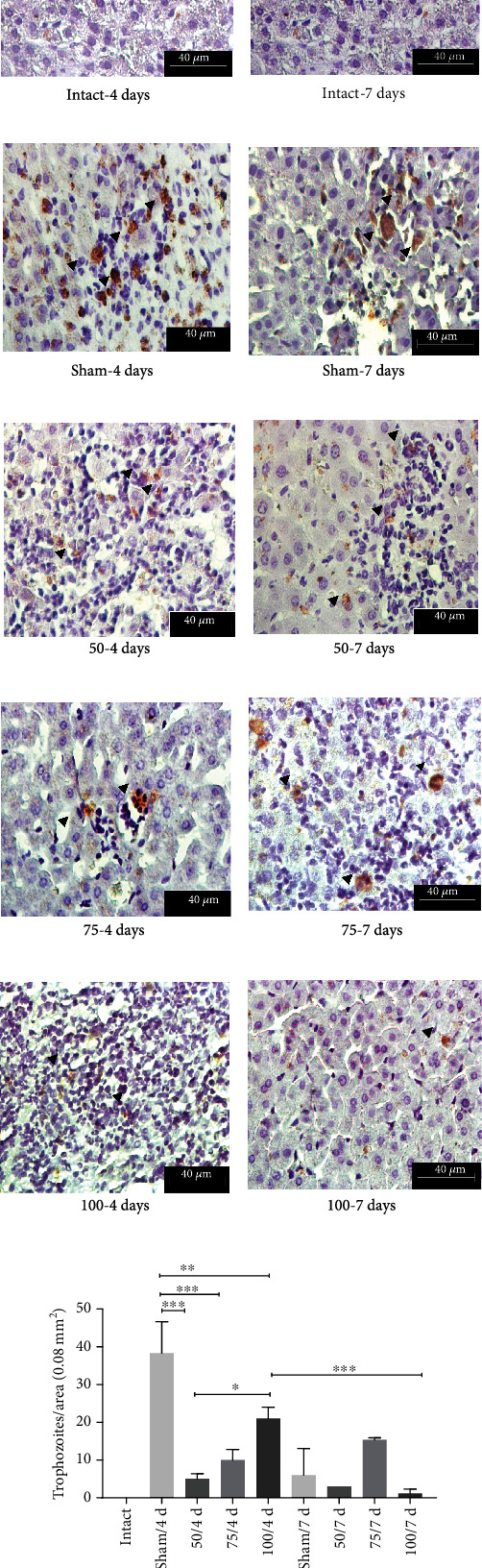
Immunohistochemistry detection for *E. histolytica* trophozoites. Original magnification 400x. (c, d) Trophozoites and their fragments (e–j) can see by the light brown color (arrowheads). The number of trophozoites was lower as increment the vaccine dose (e–j). (k) Graphical expression of quantity of *E. histolytica* trophozoites per tissue area (^∗^*p* < 0.05, ^∗∗^*p* < 0.01, ^∗∗∗^*p* < 0.001).

We observed in the sham group the abundant trophozoites in liver parenchyma (arrowheads in Figures 9(c) and 9(d)); however, in hamsters immunized with 50 *μ*g, fragments of trophozoites were identified (light brown, arrowheads in Figures 9(e) and 9(f)); in hamsters immunized with 75 *μ*g, very few trophozoites invaded by inflammatory cells (arrowheads in Figures 9(g) and 9(h)) can be identified; likewise, in hamsters immunized with 100 *μ*g, at 4 days, small areas of inflammatory infiltrate are observed with few fragments of trophozoites (arrowheads in Figure 9(i)); finally, at 7 days, the tissue liver is seen healthy, and fragments of trophozoites are seen sporadically (arrowhead in Figure 9(j)).

## 4. Discussion

Amebiasis is a neglected disease that requires a solution, having widespread prevalence, and a significant annual mortality. The main treatment (nitroimidazole) for invasive amebiasis has serious adverse effects and in many cases requires the complement of additional medications. The vaccines elaborated to date induce only partial protection of the acquired immunity of the host, and the relative importance of mucosal, cellular, and humoral immunity in protection is still undetermined [18].

The amebic antigen most frequently investigated for the development of a vaccine is the galactose-binding lectin. Vaccines based on the native or recombinant form of the Gal/GalNAc lectin proteins are the most promising, with reports of success in protecting animals against intestinal amebiasis and ALA [7, 19–21]. Clinical trials will be required to validate its efficacy in humans [18].

The aim of the present study was to test a recombinant vaccine based on the Gal-lectin antigen in a hamster model. This vaccine has better immunostimulatory characteristics [22, 23]. The most important findings of the current contribution in regard to the PE*Δ*III-LC3-KDEL3 vaccine are its effective liver tissue protection and ability to inhibit important amebic virulent functions. The latter is related to the stimulation of antibody production and the inhibition of the inflammatory response.

In response to the vaccine, the animals generated IgG-type antibodies in serum. The lectin Gal/GalNAc is by itself a highly antigenic molecule [24] that promotes the production of specific antibodies against *E. histolytica* in gerbil and mouse models of amebiasis [16]. The antibodies elicited by the vaccine were able to inhibit the cytotoxicity of virulent *E. histolytica* on HepG2 cells. This effect is especially important because the adhesion of trophozoites to host cells is a prerequisite for their capacity to destroy cells [17, 25]. A hallmark of *E. histolytica*-induced damage to host tissue is the presence of excessive inflammation, which is triggered by the activation of transcriptional factors that elicit the production and release inflammatory mediators [26–28]. For instance, TNF-*α* and IL-1*β* stimulate an inflammatory response and contribute to tissue damage [29, 30]. Additionally, TNF-*α* foments the migration of trophozoites [31]. In the current study, all animals immunized with PE*Δ*III-LC3-KDEL3 showed an attenuation of inflammatory factors in the liver microenvironment.

PE*Δ*III-LC3-KDEL3 herein promoted IL-10 cytokine gene expression, which may contribute to protection. For example, this cytokine with powerful anti-inflammatory properties [32] is related to the resistance of animals to an invasive *E. histolytica* infection by avoiding damage to host tissue and maintaining tissue homeostasis [33, 34] limiting like this an excessive inflammation, thus protecting the hamsters from ALA formation. Accordingly, there was an increase for FOXP3 in immunized groups; as a consequence, it was a decrease for NF-*κ*B.

On the other hand, some studies focusing on animal models and on human infection have established that amebiasis vaccines require a Th1 response [35]. Our results evidence an elevated expression of the IFN-*γ* gene at the last period analyzed (at 7 days). IFN-*γ* reportedly plays an important role in the host defense against *E. histolytica* [36, 37]. One mechanism described in the literature is its activation of macrophages to produce reactive oxygen species (ROS) and reactive nitrogen species (RNS), which are cytotoxic to the parasite [38].

The ALA lesion induced in the animals was minimal. This panorama was also observed by Meneses-Ruiz [39], who described “sterile protection against ALA.” The protection provided by the PE*Δ*III-LC3-KDEL3 vaccine likely stems in part from its upregulation of IFN-*γ*, leading to an effective Th1 response against *E. histolytica* and the downregulation of the immune response through IL-10 and FOXP3.

This vaccine represents a successful example of a recombinant protein that utilizes domains of a bacterial toxin for the development a potent vaccine against *E. histolytica* [16, 40]. PE*Δ*III-LC3-KDEL3 was presently administered in the absence of adjuvants, unlike the majority of studies on vaccine candidate proteins, including serine-rich protein (SREHP) [41], Gal/GalNac lectin [19], 112 kDa [42], and peroxyredoxin [43]. Many authors have reported that adjuvants induce focal necrosis and a granulomatous inflammatory response, with the predominance of macrophages at the injection site (elicited by Freund's adjuvant) [44, 45].

## 5. Conclusions

PE*Δ*III-LC3-KDEL3 recombinant protein prevent invasive amebiasis, inhibiting an excessive inflammatory response and activate a protective response in a short time.

Further research is underway to attain a more in-depth understanding of the immunological activity of this vaccine with the aim of allowing for its use in clinical trials.

## Figures and Tables

**Table 1:** Primers used for RT-qPCR.

Target	Oligonucleotides		Amplicon size
	Sense	Antisense	
IL-10	CAACTGCAGCGCTGT CATCGATTT	AGTGCCTTGAAGACG CCTTTCTCT	175
IL-1*β*	TTT CCA CAG CGA TGA GAA TG	GCCACAATGACTGAC ACCAC	217
IFN-*γ*	CAGCAGCATGGAAAA ACTGA	GCTCGCCAGAATGTT TTTGT	220
NF-*κ*B	CAGGAGCCTCAAACC TGAAG	CGTCTGTG GAGAGA AGTCC	174
FoxP3	AAGTCCTGGCCACAT CTACG	GTCTGTGCCATT TCCCA CT	246
TNF-*α*	CCTCCTGTCCGCCAT CAAG	CACTGAGTCGGTCAC CTTTC	246
*β*-Actin	TGTCACCAACTGGGA CGATA	GGGGTGTTGAAGGTC TCAAA	120

## Data Availability

The data used to support the findings of this study were supplied by Sandra Luz Martínez-Hernández under license and so cannot be made freely available. Requests for access to these data should be made to Sandra Luz Martínez-Hernández, email: lilith3050@hotmail.com. Previously reported data for design amoebic recombinant vaccine were used to support this study and are available at DOI 10.1007/s10529-017-2341-2. These prior studies are cited at relevant places within the text as references [# 9].
